# Bioluminescence in vivo imaging of autoimmune encephalomyelitis predicts disease

**DOI:** 10.1186/1742-2094-5-6

**Published:** 2008-02-01

**Authors:** Jian Luo, Peggy Ho, Lawrence Steinman, Tony Wyss-Coray

**Affiliations:** 1 Department of Neurology and Neurological Sciences, Stanford University School of Medicine, Stanford, California 94305, USA; 2 GRECC, VA Palo Alto Health Care System, Palo Alto, California 94304, USA

## Abstract

**Background:**

Experimental autoimmune encephalomyelitis is a widely used animal model to understand not only multiple sclerosis but also basic principles of immunity. The disease is scored typically by observing signs of paralysis, which do not always correspond with pathological changes.

**Methods:**

Experimental autoimmune encephalomyelitis was induced in transgenic mice expressing an injury responsive luciferase reporter in astrocytes (GFAP-luc). Bioluminescence in the brain and spinal cord was measured non-invasively in living mice. Mice were sacrificed at different time points to evaluate clinical and pathological changes. The correlation between bioluminescence and clinical and pathological EAE was statistically analyzed by Pearson correlation analysis.

**Results:**

Bioluminescence from the brain and spinal cord correlates strongly with severity of clinical disease and a number of pathological changes in the brain in EAE. Bioluminescence at early time points also predicts severity of disease.

**Conclusion:**

These results highlight the potential use of bioluminescence imaging to monitor neuroinflammation for rapid drug screening and immunological studies in EAE and suggest that similar approaches could be applied to other animal models of autoimmune and inflammatory disorders.

## Background

Experimental autoimmune encephalomyelitis (EAE) is the most commonly used animal model to study multiple sclerosis (MS), a progressive paralytic disease characterized by inflammation of the central nervous system (CNS), myelin destruction, and axonal loss [1]. EAE has proven to be an invaluable tool for the development of therapeutic approaches to MS. The model has also helped in the discovery of numerous cytokines and chemokines and the characterization of T helper cell subsets, thus playing a key role in understanding basic principles of immune function and autoimmunity [2]. Disease onset and severity of EAE is typically assessed by clinical evaluation and less frequently by postmortem pathological examination of the brain and spinal cord. The active lesion in EAE is characterized by a perivascular and parenchymal inflammatory response comprising infiltrated lymphocytes and macrophages as well as activated microglia and astrocytes. While clinical scoring is a convenient non-invasive way to assess neurological deficits, it does not always reflect pathological changes or provide direct information about cellular or molecular processes [3]. On the other hand, pathological endpoints require sacrificing animals, which can then not be followed anymore, leading to large cohorts and making it often difficult to study disease modifiers with subtle effects.

Bioluminescence imaging has been used recently to monitor and quantify gene activity repeatedly in the same animal and to study disease progression in peripheral organs with great success [4,5]. Bioluminescence imaging is quantitative and can faithfully report gene activation if appropriate promoter elements are used [6,7]. To gain molecular information in living mice about the CNS injury response in EAE, we took advantage of the fact that astrocytes react to CNS injury by increasing the transcription of glial fibrillary acidic protein (GFAP) [8]. Increased GFAP immunoreactivity coincides with onset of clinical symptoms and inflammation in acute EAE [9], and increased GFAP mRNA levels correlate with EAE symptoms in acute [10] and chronic relapsing EAE [11]. To quantify GFAP transcriptional responses in vivo we used GFAP-luciferase (GFAP-luc) transgenic mice expressing luciferase under the transcriptional control of the mouse GFAP promoter [12]. These mice have been previously used to demonstrate activation of the reporter after kainate injury [12] or to monitor host response in a mouse model of meningitis [13], but no correlation with brain injury or neuropathology was reported.

## Methods

### Mice

GFAP-luc mice [12], originally generated on the FVB/N genetic background, were crossed with C57BL/6J-Tyr^*c*-2*J *^and F1 offspring were used for experiments. Mice were between 8 and 12 weeks of age when experiments were initiated. Animal handling was performed in accordance with institutional guidelines and approved by the local IACUC.

### EAE induction and clinical assessment

MOG_35–55 _peptide (MEVGWYRSPFSRVVHLYRNGK) was synthesized by the Stanford Protein and Nucleic Acid Biotechnology Facility and purified by high-performance liquid chromatography to greater than 95% purity. Mice were immunized subcutaneously with 100 μg of MOG_35–55 _peptide emulsified in complete Freund's adjuvant (CFA) and received an intravenous (i.v.) injection of 400 ng of pertussis toxin (List Biological Laboratories, Inc., Campbell, CA), at the time of immunization and 48 h later. Mice were examined daily for clinical signs of EAE and scored as follows: 0, no paralysis; 1, loss of tail tone; 2, hindlimb weakness; 3, hindlimb paralysis; 4, hindlimb and forelimb paralysis; 5, moribund or dead.

### Bioluminescence imaging

Bioluminescence was detected with the *In Vivo *Imaging System 100 (IVIS; Xenogen, Alameda, CA) [14,15] which consists of a cooled charged coupled device (CCD) camera mounted on a dark box. Mice were injected intraperitoneally with 150 mg/kg D-luciferin (Xenogen) 10 min before imaging and anesthetized with isofluorane during imaging. Imaging signal was quantitated as photons/s/cm^2^/steridian (sr) using LIVINGIMAGE software (version 2.50) (Xenogen) and integrated over 3 min. For signal quantification, photons were obtained from a region of interest which was kept constant in area and positioning within all experiments. For longitudinal comparison of bioluminescence, baseline imaging was performed 24 h before EAE was initiated. Bioluminescence was expressed as fold induction over baseline levels. In addition, a background bioluminescence reading obtained in non-transgenic mice injected with D-luciferin was subtracted from all values.

### Tissue preparations

Mice were anesthetized with 400 mg/kg chloral hydrate (Sigma-Aldrich) and transcardially perfused with 0.9% saline. Brains and spinal cords were removed and fixed for 24 h in 4% paraformaldehyde and cryoprotected in 30% sucrose. Brains were sectioned sagittally and spinal cords were cut transversely at 40 μm using a freezing microtome (Leica, Allendale, NJ) and stored in cryoprotective medium.

### Immunohistochemistry

Immunohistochemistry was performed on free-floating sections following standard procedures [15]. Primary antibodies were against: GFAP (1:1000, Dako, Carpinteria, CA), CD68 (1:50, Serotec, Raleigh, NC), and CD4 (1:100; BD Biosciences, San Diego, CA). Primary antibody staining against CD68 and CD4 was revealed using biotinylated secondary antibodies and the ABC kit (Vector, Burlingame, CA) with Diaminobenzidine (DAB, Sigma-Aldrich). Primary antibody staining against GFAP was revealed using fluorescent secondary antibody. Quantification of the percent area covered by immunostaining was performed by Metamorph software (Molecular Devices, Sunnyvale, CA) [15].

### Statistical analysis

Data were expressed as mean ± SEM. Statistical analyses were performed with Prism 4.03 software (GraphPad Software, San Diego, CA). Correlation coefficients were calculated using Pearson correlation analyses.

## Results and discussion

To develop a model for simple non-invasive imaging of EAE we used a reporter mouse in which the injury-responsive GFAP promoter is fused to luciferase [12]. GFAP-luc mice were originally generated on the FVB/N genetic background and although these mice develop relapsing EAE with full-length myelin basic protein [16] or MOG_1–25 _[17] they did not develop comparable EAE with the H2^d ^restricted MOG_35–55 _epitope in our initial experiment (data not shown). We therefore crossed GFAP-luc mice first with C57BL/6J-Tyr^*c*-2*J *^and F1 offspring was used in all experiments herein. We immunized these mice with MOG_35–55 _emulsified in complete Freund's adjuvant (CFA) and pertussis toxin (PT). A typical clinical course was established and all immunized mice developed EAE. The first clinical signs appeared 10.5 ± 0.3 days post-immunization (dpi). Clinical scores reached a peak at 14 ± 2.6 dpi and the average duration of the peak was 6.8 ± 1.9 days.

**Figure 1:**
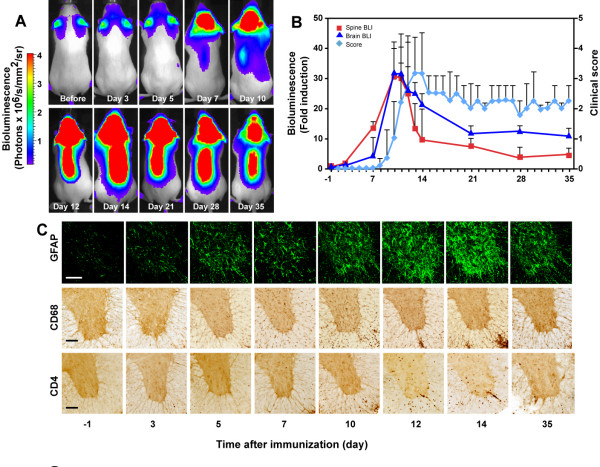
Bioluminescence imaging of GFAP-dependent transcription in EAE. EAE was induced in GFAP-luc mice with MOG_35–55 _emulsified in CFA plus pertussis toxin and bioluminescence was recorded in living mice injected with luciferin (150 mg/kg) 1 day before (-1) and at indicated time points after immunization. (**a**) Time course of bioluminescence recorded in a representative mouse. (**b**) Bioluminescence was expressed as fold induction and plotted with clinical score (n = 23). (**c**) Time course of EAE-associated neuropathology. GFAP-luc mice were sacrificed at indicated time points. Neuroinflammation was assessed by immunohistochemistry as a function of astrogliosis (GFAP), microgliosis (CD68) and T lymphocyte infiltration (CD4). Scale bar = 100 μm.

**Figure 2:**
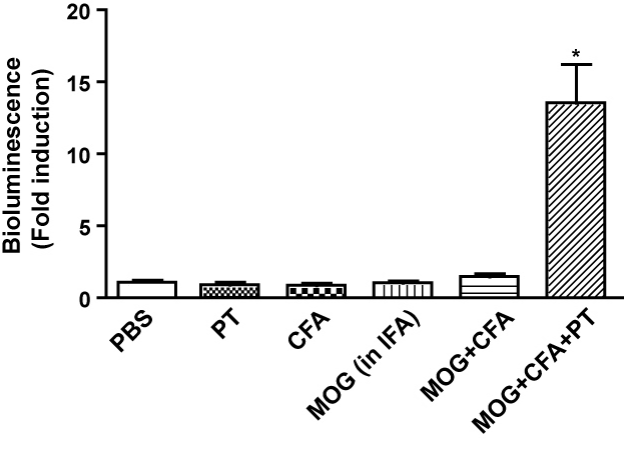
Astrocyte activation in response to adjuvant or MOG_35–55 _peptide. GFAP-luc mice were injected with PBS, CFA, PT, MOG_35–55 _peptide or combinations. Bioluminescence was recorded in living mice 1 day before and day 14 after injection and expressed as fold induction over baseline. Bars are mean ± SEM (n = 3–4 mice per group). *: *P *< 0.01 as compared with PBS by ANOVA and Tukey's post-hoc test.

Intraperitoneal injection of luciferin into immunized GFAP-luc mice resulted in a small but detectable increase in bioluminescence in the brain as early as 3 dpi and to a significant increase at 7 dpi (Figure 1A). Importantly, mice did not show any clinical signs of disease until 11 dpi (Figure 1B), consistent with rapid activation of astrocytes during EAE and astrocyte activation preceding clinical manifestations by several days [10,18]. Similar results were also obtained in the spinal cord (Figure 1A, B). The bioluminescence signal in both the brain and spinal cord peaked around 14 dpi and slowly declined throughout the remainder of the time course, with several-fold increases over baseline still detected at 35 dpi. As expected, none of the animals injected with CFA or PT alone developed clinical EAE disease and no significant increase of bioluminescence was observed (Figure 2). Overall, the relative changes in bioluminescence measured over the brain or spinal cord in immunized mice correlated strongly and significantly with clinical scores and weight loss (Table 1). These results show that GFAP-dependent reporter gene activity precedes clinical signs and can be used as a simple surrogate marker to monitor disease onset and progression in EAE.

**Table 1:** Correlation between bioluminescence and disease progression (day 1–35).

	Bioluminescence (fold induction)			
	
	Brain		Spinal cord	
	
	***R***	***P***	***R***	***P***
Clinical score	0.715	< 0.0001	0.868	< 0.0001
Weight loss	0.701	0.0001	0.813	< 0.0001
Astrogliosis (GFAP)	0.801	< 0.0001	0.858	< 0.0001
Microgliosis (CD68)	0.844	< 0.0001	0.828	< 0.0001
T cell infiltration (CD4)	0.831	< 0.0001	0.855	< 0.0001

GFAP-luc mice were immunized with MOG_35–55 _emulsified in CFA plus pertussis toxin. Mice were evaluated for clinical signs and sacrificed at 3- to 7-day interval (*n *= 3–8 mice at each time point; n = 23 mice in total). Bioluminescence was recorded in living mice after immunization and expressed as fold induction over baseline measured 1 day before immunization. Neuroinflammation was measured by immunolabeling for GFAP (astrogliosis), CD68 (microgliosis) and CD4 (T cell infiltration). These markers were quantified separately in brain (cerebellum) and spinal cord using Metamorph image analysis software. The correlation between these markers and corresponding bioluminescence was assessed by Pearson correlation analyses. *R*: correlation coefficient.

To determine whether luciferase reporter activity is also an indicator of neuropathological changes, GFAP-luc mice were evaluated for clinical signs of EAE, imaged for bioluminescence, and sacrificed at 3- to 7-day intervals during the course of EAE. Immunostaining of brain and spinal cord sections with established markers of neuroinflammation revealed typical pathological signs (Figure 1C). Activation of astrocytes (GFAP) and microglia (CD68) was observed as early as 3–7 dpi in the spinal cord, several days before clinical symptoms appear, and peaked at 14 dpi, consistent with bioluminescence imaging (Figure 1B). Indeed, bioluminescence in brain and spinal cord correlated remarkably well with astrogliosis and microgliosis (Table 1). Bioluminescence also correlated with CD4^+ ^T cell infiltration although significant T cell numbers were not seen until 10 dpi, immediately before clinical symptoms were noticeable (Figure 1C).

**Figure 3:**
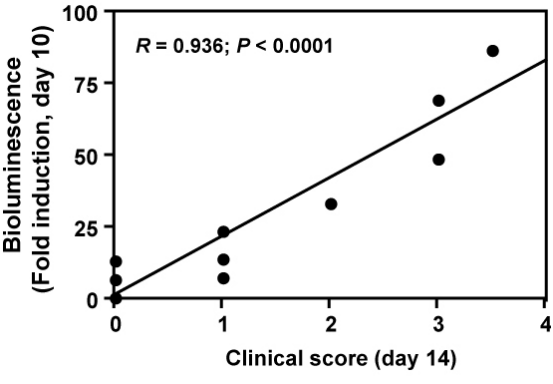
Bioluminescence recorded at day 10 correlates strongly with clinical score at day 14. GFAP-luc mice were immunized with MOG_35–55 _emulsified in CFA plus pertussis toxin. Bioluminescence was recorded in living mice and expressed as fold induction over baseline measured 1 day before immunization. Each dot represents one mouse. The correlation was assessed by Pearson correlation analysis (n = 10 mice). *R*: correlation coefficient.

While clinical scores correlated strongly with weight loss, correlations were weaker with astrogliosis and microgliosis (Table 2). The advantage of using GFAP-luciferase dependent bioluminescence imaging over clinical scoring was particularly evident in the early stages of disease. Thus, bioluminescence showed highly significant correlations with glial cell activation during the first 10 days of the disease while clinical scores did not correlate at all (Table 3). Moreover, bioluminescence but not clinical scores at early time points could predict peak clinical scores. For example, bioluminescence at 10 dpi was highly predictive of disease severity at day 14, typically the peak of disease (Figure 3).

**Table 2:** Correlation between clinical score and disease progression (day 1–35).

	Clinical score			
	
	Brain		Spinal cord	
	
	***R***	***P***	***R***	***P***
Astrogliosis (GFAP)	0.700	0.0001	0.817	< 0.0001
Microgliosis (CD68)	0.642	0.0007	0.803	< 0.0001
T cell infiltration (CD4)	0.784	< 0.0001	0.855	< 0.0001

Weight loss	***R ***= 0.855; ***P ***= 0.0001			

GFAP-luc mice were immunized with MOG_35–55 _emulsified in CFA plus pertussis toxin. Mice were evaluated daily for clinical signs. Bioluminescence was recorded in living mice and expressed as fold induction over baseline measured 1 day before immunization. Neuroinflammation was measured by immunolabeling for GFAP (astrogliosis), CD68 (microgliosis) and CD4 (T cell infiltration). These markers were quantified separately in brain (cerebellum) and spinal cord using Metamorph image analysis software. The correlation between these markers and corresponding clinical score was assessed by Pearson correlation analysis (n = 23 mice). *R*: correlation coefficient.

**Table 3:** Clinical score does not correlate with pathology in the early stages of disease (day 1–10).

	Bioluminescence (fold induction)				Clinical score			
	
	Brain		Spinal cord		Brain		Spinal cord	
	
	***R***	***P***	***R***	***P***	***R***	***P***	***R***	***P***
Astrogliosis (GFAP)	0.704	0.015	0.713	0.010	0.120	0.298	0.233	0.501
Microgliosis (CD68)	0.886	<0.0001	0.478	0.141	0.352	0.298	0.040	0.914
T cell infiltration (CD4)	0.549	0.081	0.774	0.004	0.388	0.247	0.472	0.147

Weight loss	0.823	0.0001	0.811	0.0001	***R ***= 0.354; ***P ***= 0.286			

Bioluminescence correlates with disease progression in the early stage (day 1–10), but clinical score does not. GFAP-luc mice were immunized with MOG_35–55 _emulsified in CFA plus pertussis toxin. Mice were evaluated for clinical signs and bioluminescence and sacrificed at day 3, 5, 7 and 10 (n = 11 mice). The same mice were part of the analysis shown in Table 1. The correlation was analyzed by Pearson correlation analysis. *R*: correlation coefficient.

These findings are consistent with previous reports that activation of astrocytes and increased GFAP mRNA levels correlate with EAE symptoms [10,11] and highlight the usefulness of bioluminescence imaging to monitor neuroinflammation in EAE and related animal models of inflammatory disorders. However, since the correlation of GFAP increase with long-term neurological deficits such as demyelination and neurodegeneration is not well established, it remains to be determined whether bioluminescence imaging can be applied for the study of these aspects of disease as well.

In summary, our study describes a new method of in vivo bioluminescence imaging to monitor and quantify neuroinflammation in an experimental mouse model of MS. There are still very few studies using bioluminescent reporters to image biological processes or disease in the brain. We recently reported the use of a minimal reporter for Smad2/3 transcription factor dependent signaling (SBE-luc) to monitor and quantify activation of a luciferase reporter gene in the brain [15]. We showed that the SBE-luc reporter gene is responsive to kainate injury and that the extent of activation correlates strongly with neuronal damage and microglial activation [15]. We also used GFAP-luc and SBE-luc mice recently to show that glial TGF-β signaling is activated early in EAE and may promote disease independent of autoimmune T cells [19]. These results, together with the current findings demonstrate that injury responsive genes can function as surrogate markers of brain injury and can be used to track and monitor disease progression in the brain. GFAP-luc mice should accelerate the study of autoimmune responses in EAE and neuroimmune responses in general. These and similar reporter mice could also be useful for *in vivo*, medium-throughput assessment of new therapeutic strategies for the treatment of MS and neuroinflammation.

## Conclusion

Bioluminescence imaging of GFAP-luc reporter mice provides a new tool to monitor disease onset and progression in living mice over time. These mice should be useful for the study of autoimmune responses in EAE and neuroimmune responses in general and for *in vivo*, medium-throughput assessment of new therapeutic strategies for the treatment of MS and neuroinflammation.

## Competing interests

The author(s) declare that they have no competing interests.

## Authors' contributions

J.L. performed the experiments, analyzed data, and drafted the manuscript. P.H. participated in experiments. L.S. provided valuable input in designing the study and writing the manuscript. T.W-C. designed the experiments, interpreted data, and drafted the manuscript. All authors read and approved the final manuscript.
